# Posterior Probability Matching and Human Perceptual Decision Making

**DOI:** 10.1371/journal.pcbi.1004342

**Published:** 2015-06-16

**Authors:** Richard F. Murray, Khushbu Patel, Alan Yee

**Affiliations:** Department of Psychology and Centre for Vision Research, York University, Toronto, Ontario, Canada; Oxford University, UNITED KINGDOM

## Abstract

Probability matching is a classic theory of decision making that was first developed in models of cognition. Posterior probability matching, a variant in which observers match their response probabilities to the posterior probability of each response being correct, is being used increasingly often in models of perception. However, little is known about whether posterior probability matching is consistent with the vast literature on vision and hearing that has developed within signal detection theory. Here we test posterior probability matching models using two tools from detection theory. First, we examine the models’ performance in a two-pass experiment, where each block of trials is presented twice, and we measure the proportion of times that the model gives the same response twice to repeated stimuli. We show that at low performance levels, posterior probability matching models give highly inconsistent responses across repeated presentations of identical trials. We find that practised human observers are more consistent across repeated trials than these models predict, and we find some evidence that less practised observers more consistent as well. Second, we compare the performance of posterior probability matching models on a discrimination task to the performance of a theoretical ideal observer that achieves the best possible performance. We find that posterior probability matching is very inefficient at low-to-moderate performance levels, and that human observers can be more efficient than is ever possible according to posterior probability matching models. These findings support classic signal detection models, and rule out a broad class of posterior probability matching models for expert performance on perceptual tasks that range in complexity from contrast discrimination to symmetry detection. However, our findings leave open the possibility that inexperienced observers may show posterior probability matching behaviour, and our methods provide new tools for testing for such a strategy.

## Author Summary

Decision making is partly random: a person can make different decisions at different times based on the same information. The theory of probability matching says that one reason for this randomness is that people usually choose the response that they think is most likely to be correct, but they sometimes intentionally choose the response that they think is less likely to be correct. Probability matching is a theory that was developed to describe how people try to predict the outcome of a partly random event, e.g., whether a patient has some medical condition, given the result of a medical test that does not provide perfectly accurate information. Recently, modified probability matching theories have been used to understand perceptual decision making, e.g., judging whether a sound and a visual flash were produced by the same event or by different events. We show that probability matching predicts that peoples’ perceptual decisions on difficult tasks are highly random and make poor use of the available information. We show experimentally that expert perceptual decisions are less random and more efficient than probability matching predicts. These findings help us understand how people perform a wide range of important real-world perceptual tasks, such as evaluating medical images and detecting targets in airport screening scans.

## Introduction

Human decision making is partly random, in the sense that a person can make different decisions on different occasions based on the same information. Probability matching is a theory of decision making that aims to account for this randomness. Suppose a person believes that response A has a 70% probability of being correct, and response B has a 30% probability of being correct. A person who exhibits probability matching chooses response A with 70% probability and response B with 30% probability. This is a surprising decision strategy, because it means that the person sometimes chooses the response that is less likely to be correct according to the available evidence. Nevertheless, many studies support probability matching as a model of decision making in cognitive tasks such as probability learning [1–2].

Probability matching originated in models of cognition, and variants of probability matching have also been used in models of perception. For example, Mamassian and Landy [3] had observers judge the three-dimensional shapes depicted in line drawings. In their model of this task, subjects used the intrinsically ambiguous shape information from line drawings along with assumptions about the statistical distribution of shapes in the real world to estimate the probabilities that a line drawing depicted an elliptical shape or a saddle shape. Subjects then used probability matching to choose their response, either “elliptical” or “saddle”. Acerbi et al. [4] call such a strategy “posterior probability matching,” because the subject matches their response probabilities to the posterior probability of each response being correct, instead of the prior (i.e., baseline) probability as in classic probability matching models. Posterior probability matching and related approaches have become increasingly common in models of perceptual decision making [3–8].

Posterior probability matching has some appealing features for models of perception. It offers an explanation of why perceptual decisions have a random component at all. Furthermore, it has no free parameters, which one might hope could explain why subjects’ decisions show a limited range of randomness across many perceptual tasks [9, 10].

However, little is known about whether posterior probability matching is consistent with well-supported models of perception that have been developed within signal detection theory, which take a very different approach to modelling randomness in decision making. Signal detection models typically assume that observers’ decisions depend both on information received from stimuli, and on random fluctuations in perceptual mechanisms, i.e., internal noise. If the same stimuli are repeated on different trials, the subject may make different responses, because the internal noise contributions may be different. A large literature supports signal detection models of perceptual and cognitive decision making [11]. Furthermore, posterior probability matching models represent an unusual mix of optimal and suboptimal behaviours: these models state that observers calculate the posterior probabilities that would enable them to make optimal responses based on the available stimulus information (e.g., maximizing payoff according to some utility function), but then instead behave suboptimally by making stochastic responses that match their response probabilities to the posterior probabilities.

Here we compare posterior probability matching and signal detection models of perceptual decision making. We begin by defining two very general classes of these models, and we examine the models’ behaviour using two psychophysical methods. First, we use the two-pass response consistency method, which quantifies the randomness in an observer’s decisions by examining how consistent decisions are across repeated presentations of identical trials [9, 12]. In a two-pass response consistency task the observer views one of two possible signals shown in noise, and attempts to identify the signal. This continues for some number of trials. The observer then sees the identical sequence of trials a second time (i.e., the same signals in the same samples of noise), without knowing that they are being repeated, and again attempts to identify the signal on each trial. The experimenter measures the proportion of correct responses, *P*_*C*_, and also the proportion of consistent responses, *P*_*A*_, i.e., the proportion of repeated trials on which the observer gives the same response twice. (Here the subscript “A” stands for “agreement”.) Burgess and Colborne [12] show how to use *P*_*C*_ and *P*_*A*_ to calculate the relative amounts of internal and external noise, *σ*_*I*_/*σ*_*E*_, in the decision variable of an observer.

As a second test of posterior probability matching models, we use ideal observer analysis, which measures an observer’s efficiency at a task by comparing the observer’s performance to the best performance that is theoretically possible on the task [13, 14].

We examine posterior probability matching and signal detection models' predictions as to how response consistency and efficiency should vary as a function of proportion correct. To test these models we compare their predictions to human observers’ behaviour on a two-pass response consistency task, and to results of previous experiments on response consistency and efficiency. In Experiment 1 we compare human observers' response consistency on repeated trials in a two alternative forced choice (2AFC) discrimination task to the predictions of posterior probability matching and signal detection models. In Experiment 2 we test a larger number of inexperienced observers on a shorter version of the same task in order to sample a wider range of observers and examine the effect of practice.

## Models

### Observer models

We examined the performance of posterior probability matching and signal detection models in a task where there are two possible signals (A and B) and two possible responses. This includes two-alternative identification tasks, and it also includes 2AFC tasks because we can take “signal A” to mean the two stimulus intervals in one order, and “signal B” to mean the other order. To make the models as general as possible, we modelled observers at the level of the decision variable instead of choosing specific visual or auditory stimuli. The decision variable was *D* = *E*+*I*, where *E* is a normal random variable representing the contribution of the stimulus to the decision variable, and *I* is a normal random variable representing internal noise. *E* had mean zero on signal A trials, mean *μ*_*E*_ on signal B trials, and standard deviation *σ*_*E*_ on both types of trials. The internal noise *I* had mean zero and standard deviation *σ*_*I*_, and was statistically independent of *E*. Signals A and B were equally likely.

We used the decision variable *D* to represent the information that the observer computes from a complete 2AFC trial. The most common model of 2AFC tasks, the difference model [11], assumes that observers compute one decision variable *D*_1_ from the first stimulus interval and another decision variable *D*_2_ from the second interval, and base their decisions on *D* = *D*_1_−*D*_2_. Our approach does not rely on the difference rule, and we do not need to consider the single-interval decision variables *D*_1_ and *D*_2_.

On each trial the model observers received a sample *d* from the decision variable *D*, and calculated the likelihoods that the value *d* would be generated on signal A and signal B trials:

$$P(d|A)=\phi (d,0,{\left(\sigma_{E}^{2}+\sigma_{I}^{2}\right)}^{1/2})$$ (1)

$$P(d|B)=\phi (d,\mu_{E},{\left(\sigma_{E}^{2}+\sigma_{I}^{2}\right)}^{1/2})$$ (2)

Here *ϕ*(*x*, *μ*, *σ*) is the normal probability density function. The observers used these likelihoods in Bayes’ theorem to find the posterior probability that the signal was A or B:

$$P(A|d)=\frac{P(d|A)P(A)}{P(d|A)P(A)+P(d|B)P(B)}=\frac{P(d|A)}{P(d|A)+P(d|B)}$$ (3)

P(B|d)=1−P(A|d) (4)

Here we have used the fact that *P*(*A*) = *P*(*B*). The posterior probability matching observer chose response A with probability *P*(*A*|*d*) and response B with probability *P*(*B*|*d*). We call this 'veridical posterior probability matching' (VPPM), because the observer makes veridical estimates of the posterior probabilities given the value of the decision variable, and uses these probabilities in the probability matching rule. In the rest of this article, when we speak of 'posterior probability matching' we mean the VPPM model unless we specify otherwise. The signal detection observer used a maximum a posteriori (MAP) decision rule, and chose the response that had the greater posterior probability, *P*(*A*|*d*) or *P*(*B*|*d*). When the goal is to maximize the number of correct responses, the MAP rule is the statistically optimal strategy in this task.

### Two-pass task with model observers

We modelled the VPPM and MAP observers in a two-pass task as follows. On the first pass of each trial, the decision variable was *D* = *E*+*I*, with *E* and *I* drawn from their respective distributions. On the second pass the external component *E* of the decision variable was the same as on the first pass, and the internal component *I* was a new, independent sample. In S1 Text we show that the posterior probability matching observer's proportion correct in this task is

$$p_{C}=\int_{-\infty }^{\infty }\frac{\phi {\left(u\right)}^{2}}{\phi (u)+\phi (u-d\prime_{D})}du$$ (5)

Here *ϕ*(*x*) is the standard normal probability density function, and *d*′_*D*_ is the signal-to-noise ratio of the decision variable *D*, $d\prime_{D}=\mu_{D}/{\left(\sigma_{E}^{2}+\sigma_{I}^{2}\right)}^{1/2}$. We also show that with *ρ* = *σ*_*I*_/*σ*_*E*_, the probability of two correct responses on repeated trials is

$$p_{CC}=(1+\rho^{-2}){\left(1+\rho^{2}\right)}^{1/2}\int_{-\infty }^{\infty }{\left(\int_{-\infty }^{\infty }\frac{\phi (u+v)}{\phi (u+v)+\phi (u+v-d\prime_{D})}\phi \left(v{\left(1+\rho^{-2}\right)}^{1/2}\right)dv\right)}^{2}\phi \left(u{\left(1+\rho^{2}\right)}^{1/2}\right)du$$ (6)

and the probability of two incorrect responses is

$$p_{II}=(1+\rho^{-2}){\left(1+\rho^{2}\right)}^{1/2}\int_{-\infty }^{\infty }{\left(\int_{-\infty }^{\infty }\frac{\phi (u+v-d\prime_{D})}{\phi (u+v)+\phi (u+v-d\prime_{D})}\phi \left(v{\left(1+\rho^{-2}\right)}^{1/2}\right)dv\right)}^{2}\phi \left(u{\left(1+\rho^{2}\right)}^{1/2}\right)du$$ (7)

The probability of consistent responses across two repeated trials is *P*_*A*_ = *P*_*CC*_+*P*_*II*_.

Green and Swets [11] show that the unbiased MAP observer's proportion correct is

$$p_{C}=\Phi (d\prime_{D}/2)$$ (8)

Here Φ(*x*) is the standard normal cumulative distribution function. In S1 Text we show that the probability of the MAP observer making two correct responses on repeated trials is

$$p_{CC}=\rho \int_{-\infty }^{\infty }\Phi {\left(\frac{d\prime_{D}}{2}{\left(1+\rho^{-2}\right)}^{1/2}-u\right)}^{2}\phi (\rho u)du$$ (9)

and the probability of two incorrect responses is

$$p_{II}=\rho \int_{-\infty }^{\infty }{\left(1-\Phi \left(\frac{d\prime_{D}}{2}{\left(1+\rho^{-2}\right)}^{1/2}-u\right)\right)}^{2}\phi (\rho u)du$$ (10)

Again, the probability of consistent responses is *P*_*A*_ = *P*_*CC*_+*P*_*II*_.

We used Eqs (5) to (10) to find the proportion correct and proportion of consistent responses for the VPPM and MAP model observers at several signal-to-noise ratios (*d*′_*D*_) and internal-to-external noise ratios (*σ*_*I*_/*σ*_*E*_). We used eight values of *d*′_*D*_, evenly spaced from 0 to 2.6. We used *σ*_*I*_/*σ*_*E*_ = 0, 1, and 2, which spans the range of internal-to-external noise ratios typically found with human observers [10]. Then, following Burgess and Colborne [12], we calculated the model observers’ apparent internal-to-external noise ratios from their proportion correct and proportion of consistent responses. Eqs (8), (9), and (10) give the proportion correct *P*_*C*_(*d*′_*D*_, *ρ*) and proportion of consistent responses *P*_*A*_(*d*′_*D*_, *ρ*) for a MAP observer as a function of *d*′_*D*_ and *ρ = σ*_*I*_/*σ*_*E*_. We found the apparent internal-to-external noise ratio for the model observers by numerically minimizing the following sum-of-squares error:

$$(\hat{{d^{\prime }}_{D}},\tilde{\rho })=\underset{\left(d\prime_{D},\rho \right)}{\text{argmin}}\;{\left(\hat{p_{C}}-p_{C}(d\prime_{D},\rho )\right)}^{2}+{\left(\hat{p_{A}}-p_{A}(d\prime_{D},\rho )\right)}^{2}$$ (11)

Burgess and Colborne’s method estimates the internal-to-external noise ratio of the decision variable of a MAP observer. Thus when we applied Burgess and Colborne’s method to the MAP model observer, we simply recovered the internal-to-external noise ratio that we had used to calculate *P*_*C*_ and *P*_*A*_ in the first place. When we applied the same method to the posterior probability matching observer, though, we did not recover the internal-to-external noise ratio that we had used to calculate *P*_*C*_ and *P*_*A*_, because this observer has an additional source of internal variability, namely the probability matching rule. We emphasize this point: we applied Burgess and Colborne’s method to the posterior probability matching model observer, even though this observer does not satisfy that method’s assumption that additive internal noise is the only source of randomness in the observer’s decisions. We did this in order to discover what internal-to-external noise ratios Burgess and Colborne’s method would attribute to a posterior probability matching observer. We then used Burgess and Colborne’s method with human observers (see below) to see whether they showed the pattern of internal-to-external noise ratios that is predicted by the VPPM or MAP models. We will use *σ*_*I*_/*σ*_*E*_ (or sometimes *ρ* for brevity) to denote the internal-to-external noise ratios that we used to calculate *P*_*C*_ and *P*_*A*_ for the model observers, and we will use $\tilde{\rho }$ to denote the apparent internal-to-external noise ratios that we calculated from *P*_*C*_ and *P*_*A*_ using Burgess and Colborne's method.

### Efficiency of probability matching

An observer’s efficiency at a task can be defined as the squared ratio of their *d*′ to the ideal observer’s *d*′: $\eta ={\left(d^{\prime }/{d^{\prime }}_{ideal}\right)}^{2}$ [15, 16]. The ideal observer is a theoretical model observer that achieves the best possible performance on the task. The VPPM observer’s proportion correct as a function of *d*′_*D*_ is given by Eq (5), and the observer is unbiased, so its sensitivity is *d*′ = 2Φ^−1^(*P*_*C*_). (However, this method of calculating *d*′ is really only meaningful when the observer chooses responses by comparing the decision variable to a criterion, and the VPPM observer does not do this. Consequently, this is the apparent *d*′ that an experimenter who assumes signal detection theory would attribute to the VPPM observer, based on its proportion correct. This is fine for our purposes, since our goal is simply to find the VPPM observer’s efficiency.) To examine the best-case scenario, we assumed that the VPPM observer had no internal noise and that the decision variable captured all relevant information from the stimulus. In this case the ideal observer’s *d*′ is the signal-to-noise ratio of the decision variable, *d*′_*ideal*_ = *d*′_*D*_. Thus the VPPM observer’s highest possible efficiency is *η* = (2Φ^−1^(*P*_*C*_)/*d*′_*D*_)^2^. We calculated the VPPM observer's efficiency with *σ*_*I*_/*σ*_*E*_ = 0 and several values of *d*′_*D*_ evenly spaced from 0.3 to 4.7.

### Experiment 1

#### Ethics statement

All experiments were approved by the Office of Research Ethics at York University (certificate e2014-274). All observers gave written informed consent.

#### Participants

Three observers participated in Experiment 1. The observers were graduate students in vision science laboratories, and they were practised at psychophysical tasks. One observer had practice in the task and knew that some trials would be repeated. The other two observers had not performed this task before and did not know that some trials would be repeated. None of the observers knew that we were testing a hypothesis concerning probability matching.

#### Procedure

Observers ran in 33 blocks of 300 trials where they made 2AFC contrast polarity discriminations between black and white Gaussian-profile bumps in visual white noise. S1 Fig and S1 Video show typical stimuli. Each trial showed two 500 ms stimulus intervals separated by a blank 1000 ms interstimulus interval. The signals were positive- and negative-contrast Gaussian-profile disks with scale constant σ = 0.055 degrees of visual angle (deg). The disks were shown in Gaussian white noise whose Weber contrast had pixelwise standard deviation 0.25 (power spectral density 43 mdeg^2^). The stimuli were 0.81 deg square (31 x 31 pixels). The black and white signals were randomly assigned to the two stimulus intervals. The observer pressed a key to indicate the order of the signals, and received auditory feedback. On each trial the white disk had peak contrast +*c* and the black disk had peak contrast −*c*, and the contrast magnitude *c* was adjusted across trials according to a 1-up 2-down staircase converging on 71% correct performance. A faint fixation point was shown continuously before and after the stimulus intervals. In each 300-trial block, trials 101–150 were repeated as trials 151–200. That is, the staircase was suspended for trials 151–200, and these trials were exact repetitions of trials 101–150. This sample size provided at least 100 repeated trials for each of four to six contrast levels, which gave a standard error of less than 0.05 for measurements of proportion correct and proportion of consistent responses. The blocks included unrepeated trials because this experiment was originally run for a different purpose [17]. Viewing distance was 1.65 m and background luminance was 65 cd/m^2^. Stimuli were shown on a Sony Trinitron G520 monitor (512 x 384 resolution, pixel size 0.755 mm, refresh rate 75 Hz).

We found each observer’s proportion correct and proportion of consistent responses at each signal level where there were at least 100 pairs of repeated trials. Using the same method as for the model observers (see Eq (11)), we calculated the observer’s apparent internal-to-external noise ratio at each signal level. We bootstrapped 95% confidence intervals for each internal-to-external noise ratio, resampling the number of correct and consistent responses 10,000 times from a binomial distribution. We discarded data points for which the upper end of the 95% confidence interval was greater than twice the value of the internal-to-external noise estimate itself. S1 Data provides data and analysis code for Experiments 1 and 2.

### Experiment 2

#### Participants

Twenty two new, psychophysically inexperienced observers ran in the same task that was used in Experiment 1. The observers were undergraduate students at York University who were recruited with posters placed around the campus and paid $15 for one 40 minute session. None were aware of the purpose of the experiment.

#### Procedure

Observers ran in 500 trials. For the first 100 trials, signal contrasts were chosen according to a 1-up 2-down staircase converging on 71% correct performance. After the first 100 trials the testing program fit a Weibull psychometric function to the trials up to that point, and used the fitted function to estimate the signal contrasts that generated 65% and 80% correct performance. These two signal contrasts were used for the remaining 400 trials, in random order. The remaining 400 trials were divided into four 100-trial, two-pass blocks: trials 101–150 were repeated as trials 151–200, trials 201–250 were repeated as trials 251–300, and so on. We found each observer’s apparent internal-to-external noise ratio at the two signal contrast levels used in the two-pass blocks, using the same methods as in Experiment 1. One observer had proportion correct and proportion agreement that indicated near-infinite apparent internal-to-external noise ratios at both signal contrast levels, so we discarded this observer’s data.

## Results

### Observer models

**Fig 1:**
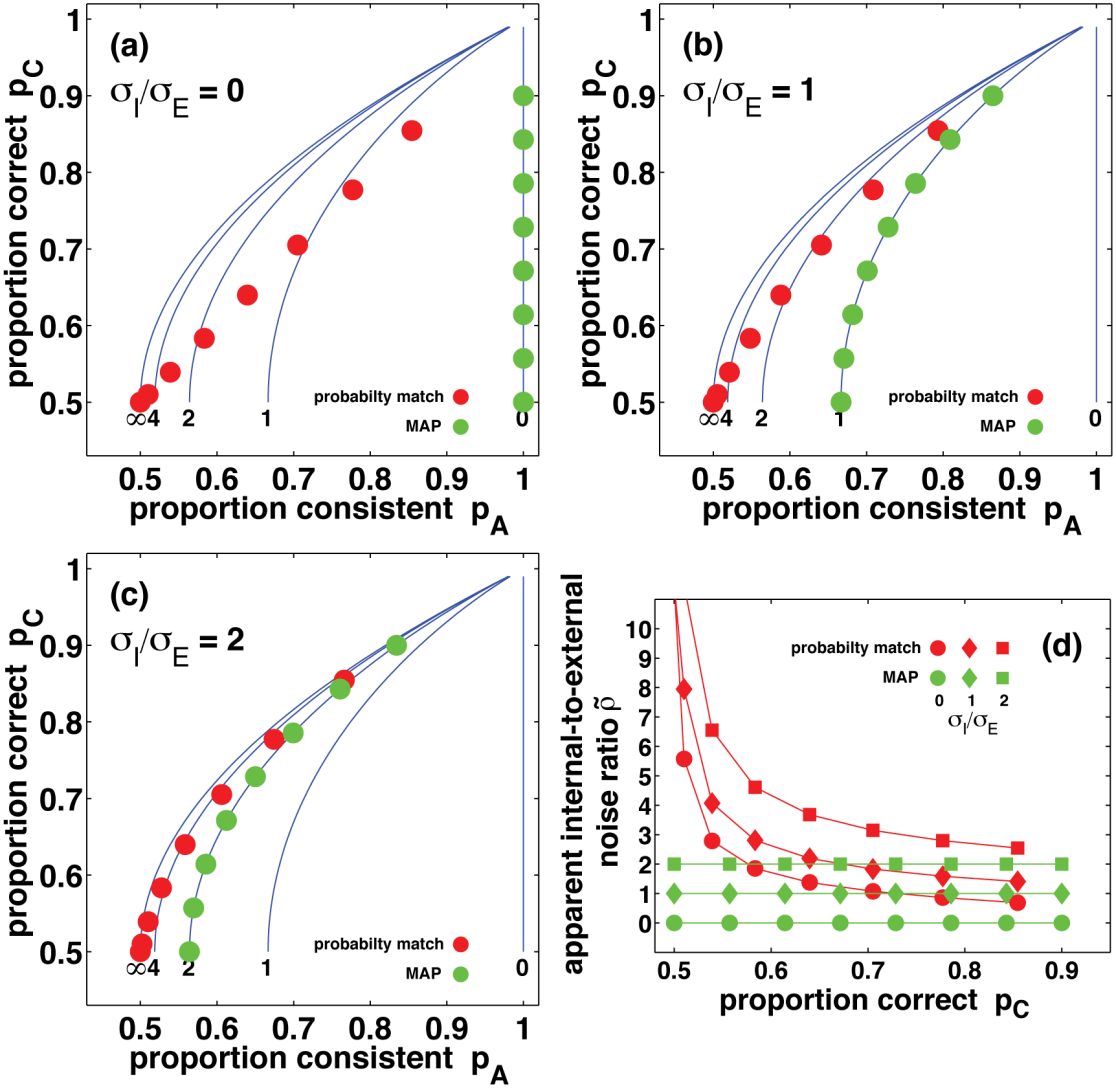
Results from two-pass experiments with model observers. In panels (a), (b), and (c) the filled circles show proportion correct and proportion of consistent responses for simulated internal-to-external noise ratios of 0, 1, and 2, respectively. Red symbols show results for the VPPM observer and green symbols show results for the MAP observer. For reference, blue lines in panels (a), (b) and (c) show the $\left(p_{A},p_{C}\right)$ contours for MAP observers with internal-to-external noise ratios of 0, 1, 2, 4, and the limit where internal noise dominates external noise (∞). Panel (d) replots data from the first three panels, showing the apparent internal-to-external noise ratio as a function of proportion correct. In panel (d), circles, diamonds, and squares show results for simulated internal-to-external noise ratios of 0, 1, and 2, respectively.

Fig 1a–1c shows the model observers’ proportion correct and proportion of consistent responses at several signal levels and internal-to-external noise ratios *σ*_*I*_/*σ*_*E*_. At any given level of signal and noise, the posterior probability matching observer has lower proportion correct than the MAP observer. Furthermore, at any given proportion correct the posterior probability matching observer’s responses are less consistent than the MAP observer’s, i.e., they have lower *P*_*A*_.

Fig 1d shows the model observers’ apparent internal-to-external noise ratios $\tilde{\rho }$, calculated from the proportion correct and proportion of consistent responses shown in Fig 1a–1c. The posterior probability matching observer has very high apparent internal-to-external noise ratios at low proportion correct, and lower but still quite high ratios at higher proportion correct. The MAP observer has constant apparent internal-to-external noise ratios across all performance levels, as expected, since as explained earlier the apparent internal-to-external noise ratio $\tilde{\rho }$ simply recovers the internal-to-external noise ratio *σ*_*I*_/*σ*_*E*_ we used to calculate the proportion correct *P*_*C*_ and the proportion of consistent responses *P*_*A*_.

### Efficiency of model observers

**Fig 2:**
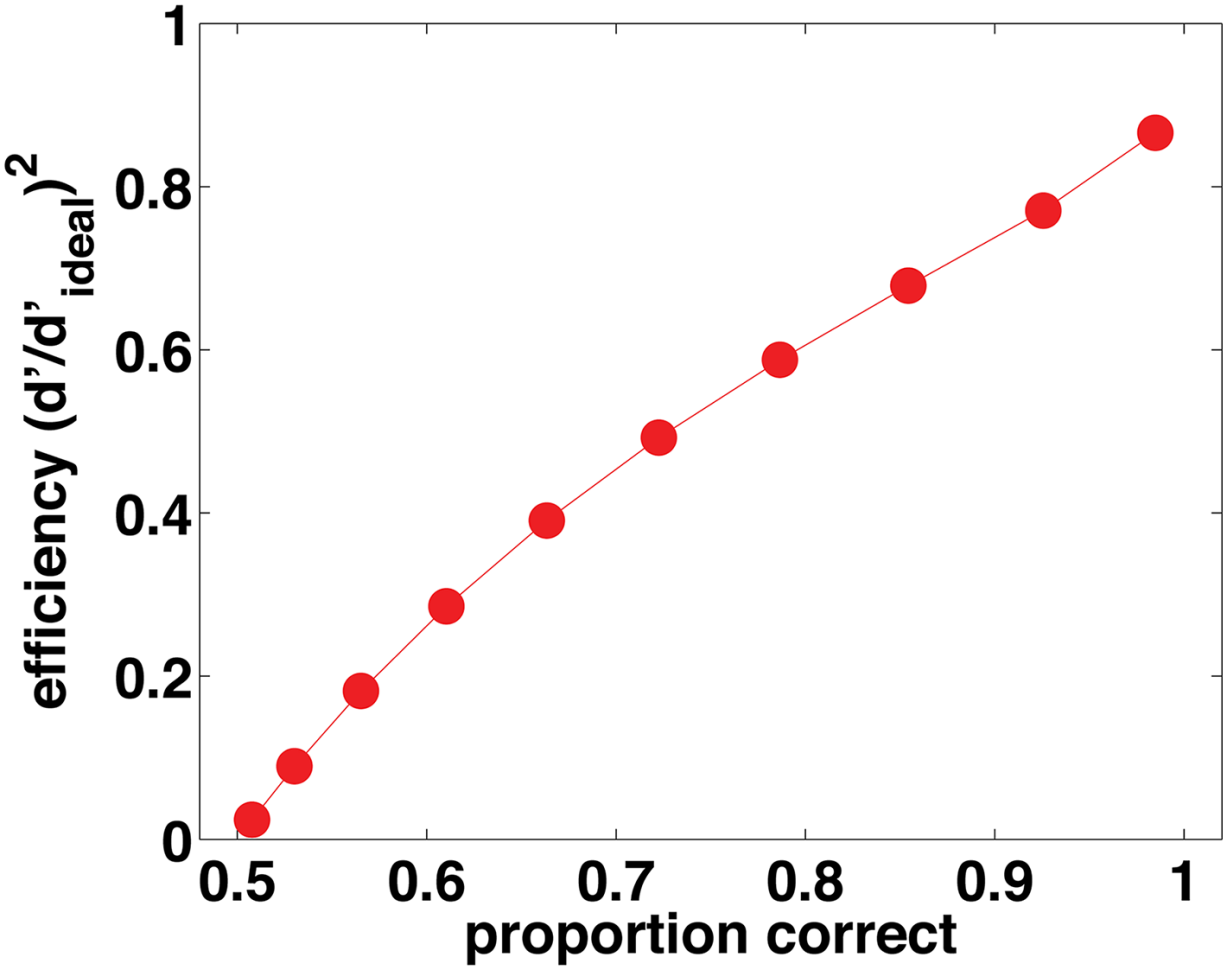
Efficiency of posterior probability matching as a function of proportion correct.

Fig 2 shows the posterior probability matching observer’s efficiency as function of proportion correct. Posterior probability matching is very inefficient at low performance levels, and even at a typical threshold performance level of 75% correct it reduces an otherwise ideal observer’s efficiency to around 50%. Efficiencies of 50% have been found in perceptual tasks at 75% threshold [13], and to be this efficient a posterior probability matching observer would have to make optimal use of the stimulus in all other respects, aside from probability matching. Furthermore, in a contrast increment detection task, Burgess, Wagner, Jennings, and Barlow [15] found efficiencies as high as 83% (standard error 15%) at 69% correct performance, and Fig 2 shows that at this proportion correct the posterior probability matching observer's maximum efficiency is 44%. Even in a task as complex as symmetry detection, Barlow [16] found efficiencies of 50% at 60% correct performance, whereas the posterior probability matching observer's maximum efficiency at this proportion correct is just 26%. Many authors have noted that probability matching is a suboptimal strategy, but perhaps it has not been realized previously how very inefficient it actually is.

In contrast, the MAP observer makes optimal use of the decision variable. To calculate the posterior probability matching observer's efficiency (Fig 2), we assumed that the internal-to-external noise ratio was zero, and that the decision variable captured all task-relevant information. Under these conditions the MAP observer is the ideal observer, so its efficiency is 100% at all values of proportion correct, and naturally no human observer can be more efficient than this.

### Experiment 1

**Fig 3:**
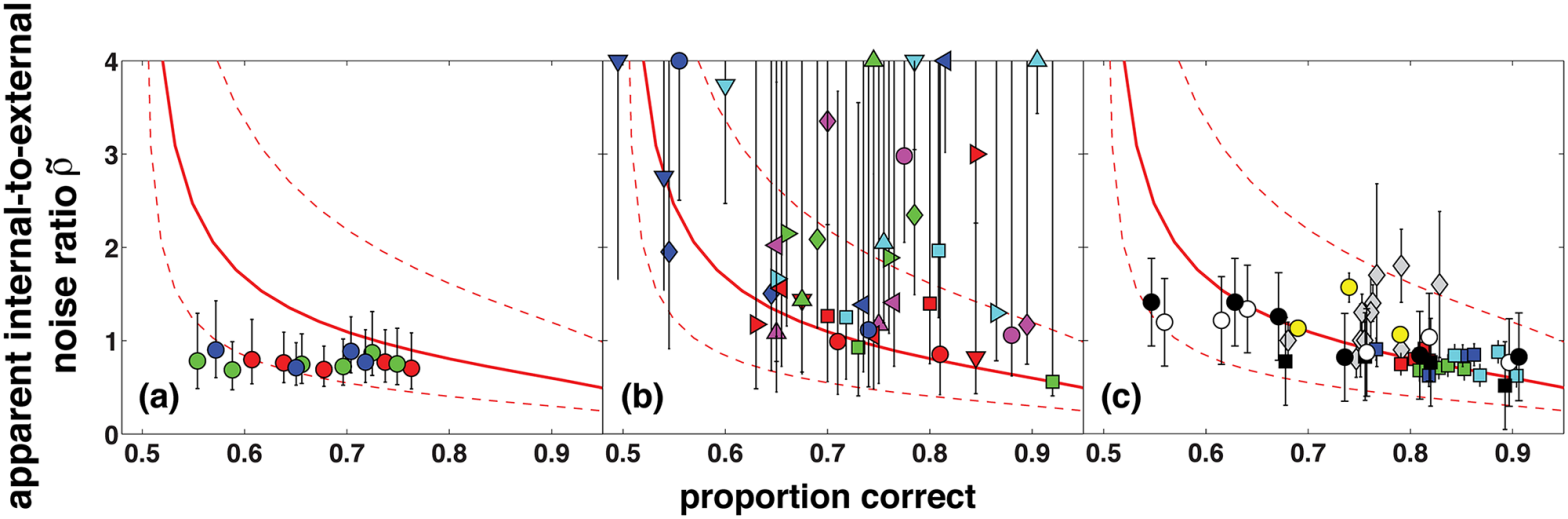
Results from two-pass experiments with human observers. In each panel the solid red line is a section of the lowest red line in Fig 1d, which represents the posterior probability matching model observer with no internal noise. The two dashed red lines have y-values twice as large and half as large as the solid red line. Error bars show 95% confidence intervals. (a) Experiment 1, practised observers. Each colour represents a different observer. (b) Experiment 2, unpractised observers. Each coloured symbol represents a different observer. (c) Data from Burgess and Colborne’s [12] Fig 2 (coloured squares) and Fig 4 (black squares, black circles, white circles), Neri’s [10] Table 1 (grey diamonds), and Murray et al.’s [17] Experiment 2 (yellow circles). For Burgess and Colborne, and for Murray et al., each data point represents a single observer. For Neri, each data point represents an average over several observers.

Fig 3a shows the results of the two-pass response consistency experiments with human observers, and also a section of the lowest red curve in Fig 1d, which represents the posterior probability matching observer with no internal noise (solid red line). For reference, the dashed red lines show internal-to-external noise ratios at twice the value and half the value of the solid red line. The observers’ apparent internal-to-external noise ratios (coloured circles) are approximately constant across performance levels, and do not show the sharp increase at low proportion correct that is predicted by the posterior probability matching model. For the three observers, least-squares linear regressions of the apparent internal-to-external noise ratio against proportion correct have slopes and bootstrapped 95% confidence intervals of -0.37 (-2.85, 2.03) (red data points), 0.28 (-1.97, 2.21) (green data points), and -0.46 (-4.24, 2.36) (blue data points). Observers' performance approximately spans the range 58% to 75% correct, and at these values the noiseless posterior probability matching observer (solid red line) has apparent internal-to-external noise ratios of 1.89 and 0.93, respectively, corresponding to a slope of -5.6, which is well outside the 95% confidence intervals of the linear regression slopes for all three observers. Furthermore, at low proportion correct (<70%) the observers’ apparent internal-to-external noise ratios are lower than is ever possible according to the posterior probability matching model. These results show decisively that observers did not follow a posterior probability matching strategy in this task.

### Experiment 2

Observers clearly did not use a posterior probability matching strategy in Experiment 1. However, only three observers ran in the experiment, and they ran in almost 10,000 trials. Previous studies on classic probability matching (i.e., prior probability matching) have found large individual differences, with only some observers showing probability matching behaviour [1, 2]. Furthermore, previous studies have found that classic probability matching behaviour decreases with practice, e.g., Shanks et al. [1] found that probability matching declined over the course of 1800 trials, with less than half the participants showing probability matching by the end of the experiment. These findings raise concerns that in Experiment 1 we may simply have chosen three observers who did not happen to exhibit probability matching behaviour, or that any probability matching behaviour may have been eliminated over the course of the experiment.

To address these concerns, in Experiment 2 we ran a larger number of observers in a shorter version of the same task. Fig 3b shows observers’ apparent internal-to-external noise ratios as a function of proportion correct. Each observer is represented by a different coloured symbol, so each coloured symbol appears twice, once for the observer’s low performance trials (at the estimated 65% threshold) and once for the observer’s high performance trials (at the estimated 80% threshold). We clipped apparent internal-to-external noise ratios at a maximum of 4.0 in order to make them visible on the plot. The internal-to-external noise ratios are higher than in Experiment 1, probably because observers were less psychophysically experienced and did not run in the task as long. Confidence intervals are also much larger than in Experiment 1, for two reasons. First, there were fewer trials, which increased the standard error of the estimates of proportion correct and proportion of consistent responses. Second, internal-to-external noise ratios were higher, and Fig 1a–1c shows that iso-*ρ* lines are closer together at higher values of *ρ*, meaning that standard errors on estimates of proportion correct and proportion of consistent responses translate into larger confidence intervals on estimates of *ρ*.

**Fig 4:**
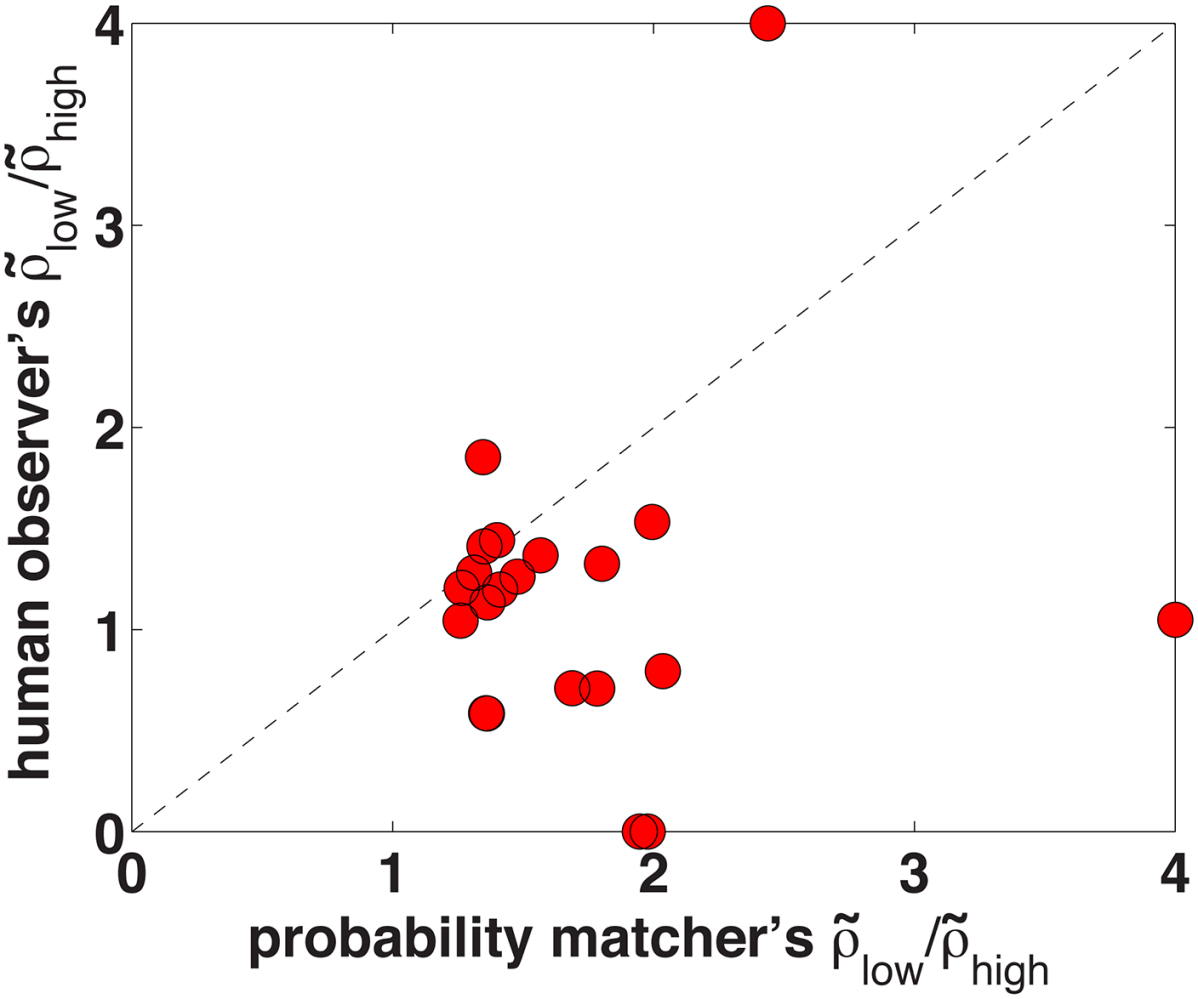
Ratio of apparent internal-to-external noise ratio at low and high performance levels. The x-axis shows the apparent internal-to-external noise ratio ${\tilde{\rho }}_{low}/{\tilde{\rho }}_{high}$ for the noiseless VPPM model and the y-axis shows the same ratio for human observers in Experiment 2.

Although the data is noisy, we can still test the VPPM model’s predictions. As explained earlier, the VPPM model predicts that apparent internal-to-external noise ratios increase sharply at low performance levels (Fig 1d). We will denote each observer’s apparent internal-to-external noise ratio at the low performance level by ${\tilde{\rho }}_{low}$, and the apparent internal-to-external noise ratio at the high performance level by ${\tilde{\rho }}_{high}$. Fig 4 shows the ratio ${\tilde{\rho }}_{low}/{\tilde{\rho }}_{high}$ for each observer, versus the ratio ${\tilde{\rho }}_{low}/{\tilde{\rho }}_{high}$ predicted by the VPPM model with no additive decision noise. We calculated the VPPM model’s predictions by looking up the predicted ${\tilde{\rho }}_{low}$ and ${\tilde{\rho }}_{high}$ for the noiseless VPPM model in Fig 1d (lowest red line), at each observer’s lower and higher proportion correct, and taking the ratio ${\tilde{\rho }}_{low}/{\tilde{\rho }}_{high}$. We clipped the ratios at a maximum of 4.0 to make them visible on the plot. Most data points in Fig 4 fall below the main diagonal, indicating that the ratio ${\tilde{\rho }}_{low}/{\tilde{\rho }}_{high}$ is not as high as predicted by the noiseless VPPM model. A sign test shows that ${\tilde{\rho }}_{low}/{\tilde{\rho }}_{high}$ is significantly lower than predicted by the noiseless VPPM model (17 of 21 data points below the diagonal, *p*<0.001).

However, we can also run less stringent tests of the VPPM model. The sign test reported in the previous paragraph tests the noiseless VPPM model, but observers may have sources of internal noise besides the posterior probability matching rule. If we re-run the sign test, comparing the actual ratio ${\tilde{\rho }}_{low}/{\tilde{\rho }}_{high}$ for human observers to the ratio predicted by the VPPM model with an internal-to-external noise ratio of *σ*_*I*_/*σ*_*E*_ = 1 (Fig 1d, middle red line), we find that the human observers’ ratio is lower than predicted in only 10 of 21 cases, which is not statistically significant according to a sign test (*p* = 0.50). Similarly, we can test the VPPM model simply by asking whether ${\tilde{\rho }}_{low}>{\tilde{\rho }}_{high}$ for human observers, as is predicted by the VPPM model with any value of *σ*_*I*_/*σ*_*E*_. The prediction ${\tilde{\rho }}_{low}>{\tilde{\rho }}_{high}$ is true for 12 of 21 observers, which is not a significant difference (*p* = 0.19). Thus our results with unpractised observers can rule out the noiseless VPPM model (which is what has been tested in previous studies of posterior probability matching in perceptual decision making), but they cannot rule out the VPPM model with additional sources of internal noise.

## Discussion

Our findings in Experiment 1 show that a broad class of posterior probability matching models is inconsistent with the results of two-pass response consistency experiments with practised human observers. Posterior probability matching predicts a sharp increase in the apparent internal-to-external noise ratio at low proportion correct that we do not find with practised observers, and at low proportion correct (<65%) it predicts apparent internal-to-external noise ratios that are higher than those we find with practised observers. Our results in Experiment 2 with less practised observers have the large measurement error associated with small numbers of trials and high internal-to-external noise ratios, but nevertheless we find evidence that internal-to-external noise ratios do not vary as much with performance level as the noiseless posterior probability matching model predicts. However, our results in Experiment 2 are consistent with VPPM models with additive Gaussian internal noise sources.

The contrast between our findings in Experiments 1 and 2 led us to examine previous two-pass studies. Fig 3c shows apparent internal-to-external noise ratios measured by Burgess and Colborne [12], Neri [10], and Murray, Bennett, and Sekuler [18]. All these studies used visual 2AFC tasks. The data points and confidence intervals representing Burgess and Colborne [12] and Murray et al. [18] show results for single observers, and those representing Neri [10] show averages across several observers. We recovered data from Burgess and Colborne using data capture software on their Figs 2 and 4, and the values from Murray et al. and Neri were given in their text (Neri’s Table 1, lines 4–8 and 14–20; Murray et al.’s results section for their Experiment 2). Fig 3c shows that most of these experiments do not span a wide range of performance levels, so they do not provide a strong test of the VPPM model’s prediction that internal-to-external noise ratios are higher at low performance levels. The most decisive evidence comes from Burgess and Colborne’s sine wave detection tasks (black circles and white circles). In these tasks the apparent internal-to-external noise ratios are only slightly higher at low performance levels than at high performance levels, and in fact at the lowest performance level (leftmost black and white circles) the internal-to-external noise ratios are much lower than is possible according to the VPPM model (solid red line).

Gold, Bennett, and Sekuler [19] found similar results in a two-pass response consistency experiment with a 1-of-10 identification task. We cannot show their data in Fig 3c, because a 1-of-10 task is not directly comparable to a 2AFC task, but Gold et al. found that apparent internal-to-external noise ratios were approximately constant over performance levels ranging from just above 10% correct to nearly 100% correct.

Our modelling results show that posterior probability matching causes a steep increase in apparent internal-to-external noise ratio at low performance levels. Burgess and Colborne [12] found that the internal-to-external noise ratio rose at low performance levels in a 2AFC sine wave detection task (black circles and white circles in Fig 3c), but not in a 2AFC disk detection task (black squares in Fig 3c). They noted that previous studies had found that intrinsic spatial uncertainty (i.e., observers being uncertain as to where a signal will appear, and so monitoring a range of possible locations) interfered much more with detection of low-contrast periodic signals such as sine waves, than with detection of low-contrast aperiodic signals such as disks. They suggested that the increase in internal-to-external noise ratios at low performance levels was due to intrinsic uncertainty. If this is correct, then we must be careful not to take an increase in apparent internal-to-external noise ratio as an unambiguous sign of posterior probability matching. In any case, this pattern in Burgess and Colborne’s data cannot be due to posterior probability matching, because as noted above, the internal-to-external noise ratios at low performance levels were much lower in their tasks than is possible according to the VPPM model.

It is worth noting that in most of the experiments we have examined, including those where we can reject the VPPM model statistically, apparent internal-to-external noise ratios are within a factor of two of the values predicted by the noiseless VPPM model, i.e., between the two dashed red lines in Fig 3a–3c. Some authors have argued that the randomness of a probability matching strategy can be adaptive, e.g., by allowing an organism or group of organisms to explore many possible courses of action, allocating effort according to the probability of success [20]. In signal detection theory, internal noise is usually thought of as an imperfection in perceptual processing. However, we speculate that under conditions where probability matching is adaptive, observers may also benefit from similar levels of randomness that are created by Gaussian internal noise and a MAP decision rule.

### Variations on posterior probability matching

Would minor adjustments to the VPPM model produce better matches to human performance? A simple argument shows that many details of the model are unimportant, and that our findings are very general. In the VPPM model, the observer calculates veridical posterior probabilities *P*(*A*|*d*) and *P*(*B*|*d*) on each trial. On trials where *P*_*C*_ = 0.5, the observer’s posterior probability estimates are *P*(*A*|*d*) = *P*(*B*|*d*) = 0.5, since any other values imply a higher proportion correct. On these trials the observer chooses responses A and B with probability 0.5, and so the proportion of consistent responses across repeated trials is also 0.5. However, *P*_*C*_ = 0.5 and *P*_*A*_ = 0.5 imply an apparent internal-to-external noise ratio of $\tilde{\rho }=\infty$ (i.e., the lower end of the blue line labelled ∞ in Fig 1a–1c passes through the point (0.5,0.5)). This means that when the VPPM observer is uncertain (*P*(*A*|*d*)≃*P*(*B*|*d*)≃0.5), it must also be highly inconsistent across repeated trials. This is very different from a MAP observer, which may be uncertain and yet highly consistent if it has little or no internal noise. Consequently, if we use Burgess and Colborne's method to interpret randomness in terms of internal noise, we must attribute a near-infinite internal-to-external noise ratio to a posterior probability matching observer as its performance approaches chance, so long as the observer bases their responses on veridical estimates of posterior probabilities.

In contrast, a non-veridical posterior probability matching observer, i.e., one that makes inaccurate estimates of *P*(*A*|*d*) and *P*(*B*|*d*), could produce very different results from those in Fig 1. Suppose that a posterior probability matching observer always assigns zero or one to *P*(*A*|*d*) and *P*(*B*|*d*), even when performing at chance, and suppose that the observer has no additive internal noise (*σ*_*I*_/*σ*_*E*_ = 0). This observer’s two-pass responses will be completely consistent, and the observer will have an apparent internal-to-external noise ratio of zero at all levels of proportion correct. It may also be possible to construct a non-veridical posterior probability matching observer that produces a more psychophysically plausible internal-to-external noise ratio. However, it is not clear what would be gained by constructing such a model, where probability matching is applied to inaccurate estimates of posterior probabilities that are, in effect, artificially chosen to avoid the high variability of posterior probability matching responses at low confidence levels. Nevertheless, this observation helps to define the range of models that our findings rule out.

### Other types of probability matching

The VPPM model should not be confused with an earlier probability matching model that was developed within signal detection theory [21–23]. In the earlier model the observer’s responses depend on whether the decision variable is greater than or less than a criterion. The observer chooses the criterion that makes their long-term response probabilities match the baseline signal probabilities, i.e., *P*(*R* = *A*) = *P*(*A*) and *P*(*R* = *B*) = *P*(*B*), instead of choosing the criterion that maximizes either the number of correct responses, or the expected payoff based on the signal probabilities and a payoff matrix. This is simply a standard signal detection model with a suboptimal criterion, and is quite different from the intrinsically random VPPM model that we have tested here and that has appeared in recent models of perceptual decision making. Furthermore, VPPM differs from prior probability matching models such as the one considered (and rejected) by Eckstein et al. [24], in which observers' response probabilities on each trial are simply matched to the baseline probabilities of the various signals, and the observer's response on a given trial does not even depend on the stimulus shown on that trial. A similar model has also been tested (and rejected) as an account of how observers distribute attention across possible signal locations [25].

It may be that people exhibit posterior probability matching in some types of perceptual tasks but not others. For example, there is at least one notable difference between our contrast discrimination task, where some observers did not exhibit posterior probability matching, and Gifford et al.’s [6] task, where model fitting suggests that they did. In our task, subjects discriminated between black-disk-first and white-disk-first stimuli, and the performance-limiting factors were the faintness of the signals and the power of the external noise. In Gifford et al.’s task, subjects categorized auditory signals of various frequencies as having been generated from one of two broad, overlapping frequency ranges. Subjects’ ability to perceive the signal frequency precisely was not the performance-limiting factor. Rather, the task was difficult because the two categories overlapped, and contained many of the same frequencies. Thus, even though this was a simple frequency categorization task, it was not limited by low-level sensory information, but instead by the fact that clearly perceived signals gave ambiguous information about the correct response. In this way it is similar to the cued probability learning tasks that have supported prior probability matching models in the past [26], i.e., models in which observers match their response probabilities to the baseline probabilities of the signals being viewed. We suggest that human observers may be more likely to show posterior probability matching in tasks where performance is limited by the ambiguity of clearly perceived signals, than in simple discrimination tasks that are limited by the perceptual signal-to-noise ratio.

In summary, posterior probability matching causes highly inconsistent responses, especially at low performance levels, and it is also inefficient. We can definitively rule out posterior probability matching models for practised observers in four typical perceptual tasks, where human observers are more consistent or more efficient than such models allow: the contrast polarity discrimination task reported here, sine wave detection [12], sine wave contrast discrimination [15], and symmetry detection [16]. We also find evidence that less practised observers are more consistent at low performance levels than noiseless posterior probability matching models predict, but these results are consistent with a posterior probability matching model with additional internal noise. We conclude that posterior probability matching is not a promising general-purpose model of expert perceptual decisions that are limited by low-level perceptual information. However, it may be viable as a theory of untrained perceptual decision making, and of decision making in tasks where performance is not limited by low-level perceptual information. The methods we have introduced also provide new tools for testing posterior probability matching models in more complex perceptual and cognitive tasks.

## Supporting Information

S1 Text Proportion correct and proportion agreement for VPPM and MAP models. (PDF)

S1 Fig Typical stimuli in Experiments 1 and 2. (TIF)

S1 Video Typical stimuli in Experiments 1 and 2. (AVI)

S1 Data Data and analysis routines. This MATLAB code reproduces the results from Experiment 1 shown in Fig 3a and the results from Experiment 2 shown in Fig 3b.
(ZIP)
