# Supplementary material for: Posterior Probability Matching and Human Perceptual Decision Making
Source: PLoS Comput Biol. 2015 Jun 16;11(6):e1004342. doi: 10.1371/journal.pcbi.1004342 (PMC4469678; doi:10.1371/journal.pcbi.1004342)
Supplement: S1 Text — (PDF) [file pcbi.1004342.s001.pdf]

**Supporting Text S1. Proportion correct and proportion agreement.**

Here we derive equations for the proportion correct and proportion of consistent responses for veridical probability matching model (VPPM) and maximum a posteriori (MAP) observers. The derivations for the MAP observer are similar to Burgess and Colborne's [12], and the derivations for the VPPM observer follow a similar approach. We give them in detail here for completeness, and in order to give all relevant results in the same notation.

Task and notation

We model observers' performance in a two-pass task where there are two possible signals (A and B) and two possible responses. This includes two-alternative identification tasks, and it also includes 2AFC tasks because we can take "signal A" to mean the two stimulus intervals in one order, and "signal B" to mean the other order. The signal *S* is a random variable that can take values *A* or *B*, and the two values are equally probable. On the first pass the observer's response is a random variable $R_1$, and on the second pass it is $R_2$. $R_1$ and $R_2$ also take values *A* or *B*.

We assume that on the first pass the observer's responses are based on a decision variable $D_1 = E + I_1$, and that on the second pass they are based on $D_2 = E + I_2$. *E* is a normal random variable that represents the contribution of the stimulus to the decision variable. *E* has mean zero on trials where $S = A$, and mean $\mu_E$ on trials where $S = B$. *E* has standard deviation $\sigma_E$ on both types of trials. $I_1$ and $I_2$ are random variables that represent internal noise. They have mean zero and standard deviation $\sigma_I$. *E*, $I_1$, and $I_2$ are mutually independent. The standard deviation of the decision variables is $\sigma_P = (\sigma_E^2 + \sigma_I^2)^{1/2}$, and their signal-to-noise ratio is $d'_D = \mu_E / \sigma_P$. This model of the decision variable can be used to describe yes-no and 2AFC

tasks [11], so the results that we derive below apply to both tasks.

In the main text we explain how VPPM and MAP observers choose their responses based on samples *d* from the decision variables $D_1$ and $D_2$, although there we did not introduce separate decision variables $D_1$ and $D_2$ for the two passes. For the VPPM observer, the probability of giving response *A* or *B* is

$$P(R_i = k \mid D_i = d) = P(S = k \mid D_i = d) \tag{S1}$$

For the unbiased MAP observer, the decision rule is

$$R_i = \begin{cases} A & \text{if } D_i < \mu_E / 2 \\ B & \text{otherwise} \end{cases} \tag{S2}$$

The VPPM observer

On the first pass of a trial the probability of the VPPM observer making a correct response is

$$p_C = P(R_1 = S) \tag{S3}$$

$$= P(R_1 = A \mid S = A)P(S = A) + P(R_1 = B \mid S = B)P(S = B) \tag{S4}$$

We can evaluate the first probability in equation (S4) by using the law of total probability to break up the probability into cases that are conditional on the value of the decision variable $D_1$.

$$P(R_1 = A \mid S = A) = \int_{-\infty}^{\infty} P(R_1 = A \mid S = A, D_1 = x)P(D_1 = x \mid S = A)\,dx \tag{S5}$$

Given the value of the decision variable $D_1$, the observer's response is independent of the the signal $S$.

$$= \int_{-\infty}^{\infty} P(R_1 = A \mid D_1 = x)P(D_1 = x \mid S = A)\,dx \tag{S6}$$

For the VPPM observer, $P(R_1 = A \mid D_1 = x) = P(S = A \mid D_1 = x)$.

$$= \int_{-\infty}^{\infty} P(S = A \mid D_1 = x) P(D_1 = x \mid S = A)\,dx \tag{S7}$$

Now we can use Bayes' theorem and the probability density of $D_1$.

$$= \int_{-\infty}^{\infty} \frac{\phi(x,0,\sigma_P)}{\phi(x,0,\sigma_P) + \phi(x,\mu_E,\sigma_P)} \phi(x,0,\sigma_P)\,dx \tag{S8}$$

Here $\phi(x,\mu,\sigma)$ is the normal probability density function. When we omit values of $\mu$ and $\sigma$ they default to zero and one, respectively, i.e., $\phi(x)$ is the standard normal probability density function. Next we use the fact that for any $\mu$ and $\sigma$, $\phi(x,\mu,\sigma) = \sigma^{-1}\phi((x-\mu)/\sigma)$.

$$= \int_{-\infty}^{\infty} \frac{\phi(x/\sigma_P)^2 \sigma_P^{-1}}{\phi(x/\sigma_P) + \phi((x-\mu_E)/\sigma_P)}\,dx \tag{S9}$$

Let $u = x/\sigma_P$, and so $du = dx/\sigma_P$.

$$= \int_{-\infty}^{\infty} \frac{\phi(u)^2}{\phi(u) + \phi(u - d'_D)}\,du \tag{S10}$$

Symmetry or an analogous derivation show that $P(R_1 = B \mid S = B)$, the third probability in line (S4), is given by the same expression as in equation (S10):

$$P(R_1 = B \mid S = B) = \int_{-\infty}^{\infty} \frac{\phi(u)^2}{\phi(u) + \phi(u - d'_D)}\,du \tag{S11}$$

$P(S = A) + P(S = B) = 1$, so equation (S4) becomes

$$p_C = \int_{-\infty}^{\infty} \frac{\phi(u)^2}{\phi(u) + \phi(u - d'_D)}\,du \tag{S12}$$

This is equation (5) in the main text. The proportion correct is the same on the second pass of a trial.

The probability of the VPPM observer making correct responses on both passes of a trial is

$$p_{CC} = P(R_1 = S, R_2 = S) \tag{S13}$$

$$= P(R_1 = A, R_2 = A \mid S = A)P(S = A) + P(R_1 = B, R_2 = B \mid S = B)P(S = B) \tag{S14}$$

We evaluate the first probability in equation (S14) using the law of total probability, breaking the probability up into cases conditional on the external contribution $E$ to the decision variable.

$$P(R_1 = A, R_2 = A \mid S = A) = \int_{-\infty}^{\infty} P(R_1 = A, R_2 = A \mid S = A, E = x)P(E = x \mid S = A)\,dx \tag{S15}$$

$$= \int_{-\infty}^{\infty} P(R_1 = A \mid E = x)P(R_2 = A \mid E = x)P(E = x \mid S = A)\,dx \tag{S16}$$

The two internal noise variables $I_1$ and $I_2$ follow the same distribution, so $P(R_1 = A \mid E = x) = P(R_2 = A \mid E = x)$, and equation (S16) becomes

$$= \int_{-\infty}^{\infty} P(R_1 = A \mid E = x)^2 P(E = x \mid S = A)\,dx \tag{S17}$$

To evaluate the squared probability in equation (S16), we use the law of total probability and condition on the internal noise variable $I_1$.

$$= \int_{-\infty}^{\infty} \left( \int_{-\infty}^{\infty} P(R_1 = A \mid E = x, I_1 = y)P(I_1 = y \mid E = x)\,dy \right)^2 P(E = x \mid S = A)\,dx \tag{S18}$$

$$= \int_{-\infty}^{\infty} \left( \int_{-\infty}^{\infty} P(S = A \mid E = x, I_1 = y)P(I_1 = y)\,dy \right)^2 P(E = x \mid S = A)\,dx \tag{S19}$$

$$= \int_{-\infty}^{\infty} \left( \int_{-\infty}^{\infty} \frac{\phi(x+y, 0, \sigma_P)}{\phi(x+y, 0, \sigma_P) + \phi(x+y, \mu_E, \sigma_P)} \phi(y, 0, \sigma_I)\,dy \right)^2 \phi(x, 0, \sigma_E)\,dx \tag{S20}$$

$$= \int_{-\infty}^{\infty} \left( \int_{-\infty}^{\infty} \frac{\phi((x+y)/\sigma_P)}{\phi((x+y)/\sigma_P) + \phi((x+y-\mu_E)/\sigma_P)} \phi(y/\sigma_I)\sigma_I^{-1}\,dy \right)^2 \phi(x/\sigma_E)\sigma_E^{-1}\,dx \tag{S21}$$

Let $u = x/\sigma_P$ and $v = y/\sigma_P$.

$$= \int_{-\infty}^{\infty} \left( \int_{-\infty}^{\infty} \frac{\phi(u+v)}{\phi(u+v) + \phi(u+v-d'_D)} \phi(v\sigma_P/\sigma_I)\sigma_P\sigma_I^{-1}\,dv \right)^2 \phi(u\sigma_P/\sigma_E)\sigma_P\sigma_E^{-1}\,du \tag{S22}$$

Let $\rho = \sigma_I / \sigma_E$.

$$= (1+\rho^{-2})(1+\rho^2)^{1/2} \int_{-\infty}^{\infty} \left( \int_{-\infty}^{\infty} \frac{\phi(u+v)}{\phi(u+v)+\phi(u+v-d'_D)} \phi(v(1+\rho^{-2})^{1/2}) dv \right)^2 \phi(u(1+\rho^2)^{1/2}) du \quad \text{(S23)}$$

Symmetry or an analogous derivation show that $P(R_1 = B, R_2 = B \mid S = B)$, the third probability in line (S14), has the same value as line (S23). $P(S = A) + P(S = B) = 1$, so line (S14) becomes

$$p_{CC} = (1+\rho^{-2})(1+\rho^2)^{1/2} \int_{-\infty}^{\infty} \left( \int_{-\infty}^{\infty} \frac{\phi(u+v)}{\phi(u+v)+\phi(u+v-d'_D)} \phi(v(1+\rho^{-2})^{1/2}) dv \right)^2 \phi(u(1+\rho^2)^{1/2}) du \quad \text{(S24)}$$

This is equation (6) in the main text.

The probability of the VPPM observer making incorrect responses on both passes of a trial is

$$p_{II} = P(R_1 \neq S, R_2 \neq S) \quad \text{(S25)}$$

$$= P(R_1 = B, R_2 = B \mid S = A) P(S = A) + P(R_1 = A, R_2 = A \mid S = B) P(S = B) \quad \text{(S26)}$$

The first probability in this expression is

$$P(R_1 = B, R_2 = B \mid S = A) = \int_{-\infty}^{\infty} P(R_1 = B, R_2 = B \mid S = A, E = x) P(E = x \mid S = A) dx \quad \text{(S27)}$$

$$= \int_{-\infty}^{\infty} P(R_1 = B \mid E = x) P(R_2 = B \mid E = x) P(E = x \mid S = A) dx \quad \text{(S28)}$$

$$= \int_{-\infty}^{\infty} P(R_1 = B \mid E = x)^2 P(E = x \mid S = A) dx \quad \text{(S29)}$$

$$= \int_{-\infty}^{\infty} \left( \int_{-\infty}^{\infty} P(R_1 = B \mid E = x, I_1 = y) P(I_1 = y \mid E = x) dy \right)^2 P(E = x \mid S = A) dx \quad \text{(S30)}$$

$$= \int_{-\infty}^{\infty} \left( \int_{-\infty}^{\infty} P(S = B \mid E = x, I_1 = y) P(I_1 = y) dy \right)^2 P(E = x \mid S = A) dx \quad \text{(S31)}$$

$$= \int_{-\infty}^{\infty} \left( \int_{-\infty}^{\infty} \frac{\phi(x+y, \mu_E, \sigma_P)}{\phi(x+y, 0, \sigma_P) + \phi(x+y, \mu_E, \sigma_P)} \phi(y, 0, \sigma_I) dy \right)^2 \phi(x, 0, \sigma_E) dx \quad \text{(S32)}$$

$$= \int_{-\infty}^{\infty} \left( \int_{-\infty}^{\infty} \frac{\phi((x+y-\mu_E)/\sigma_P)}{\phi((x+y)/\sigma_P) + \phi((x+y-\mu_E)/\sigma_P)} \phi(y/\sigma_I)\sigma_I^{-1}\, dy \right)^2 \phi(x/\sigma_E)\sigma_E^{-1}\, dx \qquad \text{(S33)}$$

Let $u = x/\sigma_P$ and $v = y/\sigma_P$.

$$= \int_{-\infty}^{\infty} \left( \int_{-\infty}^{\infty} \frac{\phi(u+v-d'_D)}{\phi(u+v) + \phi(u+v-d'_D)} \phi(v\sigma_P/\sigma_I)\sigma_P\sigma_I^{-1} dv \right)^2 \phi(u\sigma_P/\sigma_E)\sigma_P\sigma_E^{-1}\, du \qquad \text{(S34)}$$

Let $\rho = \sigma_I/\sigma_E$.

$$= (1+\rho^{-2})(1+\rho^2)^{1/2} \int_{-\infty}^{\infty} \left( \int_{-\infty}^{\infty} \frac{\phi(u+v-d'_D)}{\phi(u+v) + \phi(u+v-d'_D)} \phi(v(1+\rho^{-2})^{1/2}) dv \right)^2 \phi(u(1+\rho^2)^{1/2})\, du \qquad \text{(S35)}$$

Symmetry or an analogous derivation show that $P(R_1 = A, R_2 = A \mid S = B)$, the third probability in line (S26), has the same value as line (S35). $P(S = A) + P(S = B) = 1$, so line (S26) becomes

$$p_{II} = (1+\rho^{-2})(1+\rho^2)^{1/2} \int_{-\infty}^{\infty} \left( \int_{-\infty}^{\infty} \frac{\phi(u+v-d'_D)}{\phi(u+v) + \phi(u+v-d'_D)} \phi\left(v(1+\rho^{-2})^{1/2}\right) dv \right)^2 \phi\left(u(1+\rho^2)^{1/2}\right) du \qquad \text{(S36)}$$

This is equation (7) in the main text.

The MAP observer

The probability of the MAP observer making a correct response is given by equation (8) in the main text, as shown by Green and Swets [11].

The probability of the MAP observer making correct responses on both passes of a trial is

$$p_{CC} = P(R_1 = S, R_2 = S) \qquad \text{(S37)}$$

$$= P(R_1 = A, R_2 = A \mid S = A)P(S = A) + P(R_1 = B, R_2 = B \mid S = B)P(S = B) \qquad \text{(S38)}$$

The first probability in this expression is

$$P(R_1 = A, R_2 = A \mid S = A) = \int_{-\infty}^{\infty} P(R_1 = A, R_2 = A \mid S = A, E = x)P(E = x \mid S = A)\, dx \qquad \text{(S39)}$$

$$= \int_{-\infty}^{\infty} P(R_1 = A \mid E = x) P(R_2 = A \mid E = x) P(E = x \mid S = A) dx \tag{S40}$$

$$= \int_{-\infty}^{\infty} P(R_1 = A \mid E = x)^2 P(E = x \mid S = A) dx \tag{S41}$$

$$= \int_{-\infty}^{\infty} P(E + I_1 < \mu_E / 2 \mid E = x)^2 P(E = x \mid S = A) dx \tag{S42}$$

$$= \int_{-\infty}^{\infty} \Phi((\mu_E / 2) - x, 0, \sigma_I)^2 \phi(x, 0, \sigma_E) dx \tag{S43}$$

Here $\Phi(x,\mu,\sigma)$ is the normal cumulative distribution function. When we omit values of $\mu$ and $\sigma$ they default to zero and one, respectively, i.e., $\Phi(x)$ is the standard normal cumulative distribution function.

$$= \int_{-\infty}^{\infty} \Phi((d'_D / 2)(\sigma_P / \sigma_I) - x / \sigma_I)^2 \phi(x / \sigma_E) \sigma_E^{-1} dx \tag{S44}$$

Let $u = x / \sigma_I$.

$$= \int_{-\infty}^{\infty} \Phi((d'_D / 2)(\sigma_P / \sigma_I) - u)^2 \phi(u \sigma_I / \sigma_E) \sigma_I / \sigma_E du \tag{S45}$$

$$= \rho \int_{-\infty}^{\infty} \Phi((d'_D / 2)(1 + \rho^{-2})^{1/2} - u)^2 \phi(\rho u) du \tag{S46}$$

Symmetry or an analogous derivation show that $P(R_1 = B, R_2 = B \mid S = B)$, the third probability in line (S38), has the same value as line (S46). $P(S = A) + P(S = B) = 1$, so line (S38) becomes

$$p_{CC} = \rho \int_{-\infty}^{\infty} \Phi((d'_D / 2)(1 + \rho^{-2})^{1/2} - u)^2 \phi(\rho u) du \tag{S47}$$

This is equation (9) in the main text.

The probability of the MAP observer making incorrect responses on both passes of a trial is

$$p_{CC} = P(R_1 \neq S, R_2 \neq S) \tag{S48}$$

$$= P(R_1 = B, R_2 = B \mid S = A) P(S = A) + P(R_1 = A, R_2 = A \mid S = B) P(S = B) \tag{S49}$$

The first probability in this expression is

$$P(R_1 = B, R_2 = B \mid S = A) = \int_{-\infty}^{\infty} P(R_1 = B, R_2 = B \mid S = A, E = x) P(E = x \mid S = A)\, dx \qquad \text{(S50)}$$

$$= \int_{-\infty}^{\infty} P(R_1 = B \mid E = x) P(R_2 = B \mid E = x) P(E = x \mid S = A)\, dx \qquad \text{(S51)}$$

$$= \int_{-\infty}^{\infty} P(R_1 = B \mid E = x)^2 P(E = x \mid S = A)\, dx \qquad \text{(S52)}$$

$$= \int_{-\infty}^{\infty} P(E + I_1 \geq \mu_E / 2 \mid E = x)^2 P(E = x \mid S = A)\, dx \qquad \text{(S53)}$$

$$= \int_{-\infty}^{\infty} (1 - \Phi((\mu_E / 2) - x, 0, \sigma_I))^2 \phi(x, 0, \sigma_E)\, dx \qquad \text{(S54)}$$

$$= \int_{-\infty}^{\infty} (1 - \Phi((d'_D / 2)(\sigma_P / \sigma_I) - x / \sigma_I))^2 \phi(x / \sigma_E) \sigma_E^{-1}\, dx \qquad \text{(S55)}$$

Let $u = x / \sigma_I$.

$$= \int_{-\infty}^{\infty} (1 - \Phi((d'_D / 2)(\sigma_P / \sigma_I) - u))^2 \phi(u\sigma_I / \sigma_E) \sigma_I / \sigma_E\, du \qquad \text{(S56)}$$

$$= \rho \int_{-\infty}^{\infty} (1 - \Phi((d'_D / 2)(1 + \rho^{-2})^{1/2} - u))^2 \phi(\rho u)\, du \qquad \text{(S57)}$$

Symmetry or an analogous derivation show that $P(R_1 = A, R_2 = A \mid S = B)$, the third probability in line (S49), has the same value as line (S57). $P(S = A) + P(S = B) = 1$, so line (S49) becomes

$$p_{II} = \rho \int_{-\infty}^{\infty} (1 - \Phi((d'_D / 2)(1 + \rho^{-2})^{1/2} - u))^2 \phi(\rho u)\, du \qquad \text{(S58)}$$

This is equation (10) in the main text.
