# Supplementary figures and images for: Posterior Probability Matching and Human Perceptual Decision Making
Source: PLoS Comput Biol. 2015 Jun 16;11(6):e1004342. doi: 10.1371/journal.pcbi.1004342 (PMC4469678; doi:10.1371/journal.pcbi.1004342)

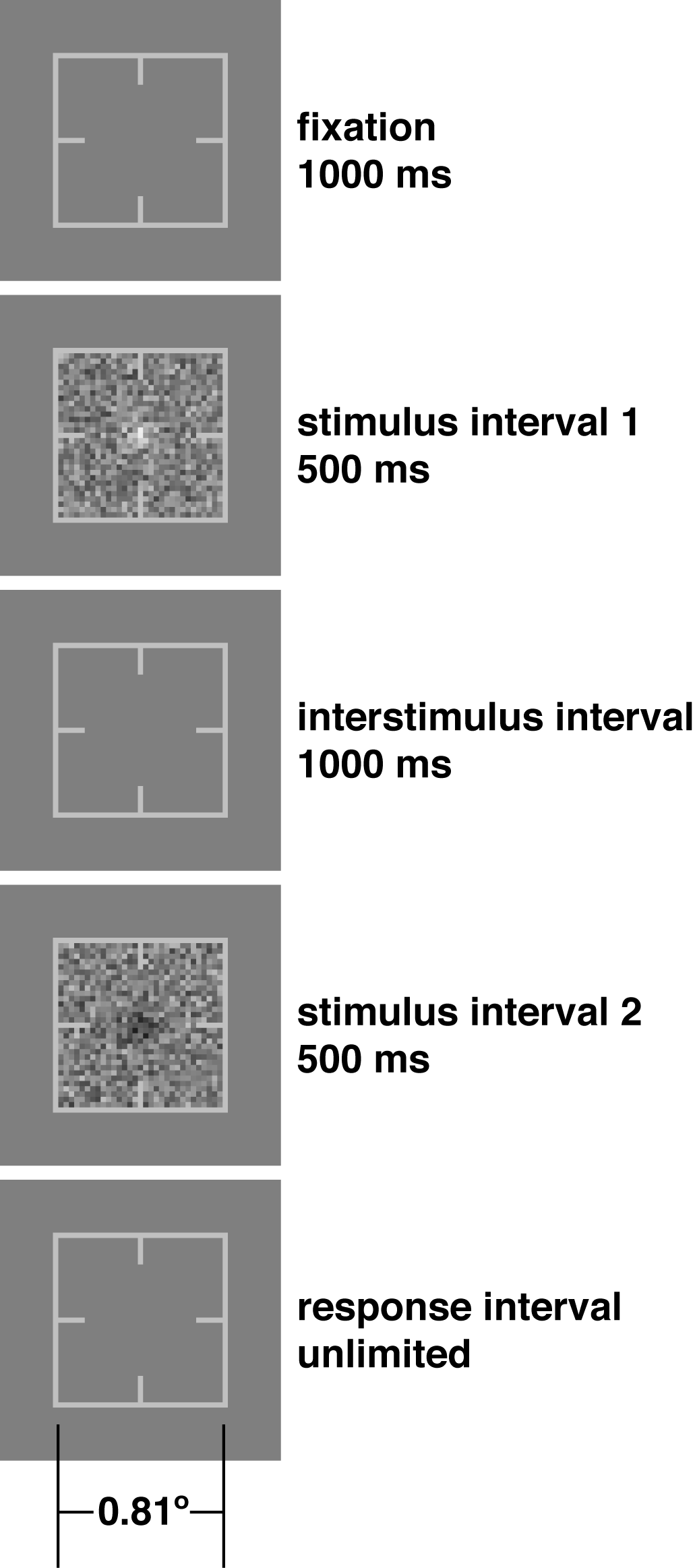

Supplement: S1 Fig — (TIF) [file pcbi.1004342.s002.tif]
